# Anthropometrics, Athletic Abilities and Perceptual-Cognitive Skills Associated With Baseball Pitching Velocity in Young Athletes Aged Between 10 and 22 Years Old

**DOI:** 10.3389/fspor.2022.822454

**Published:** 2022-03-29

**Authors:** Mathieu Tremblay, Charles Tétreau, Laurie-Ann Corbin-Berrigan, Martin Descarreaux

**Affiliations:** Groupe de Recherche sur les Affections Neuromusculosquelettiques, Département des sciences de l'activité physique, Université du Québec à Trois-Rivières, Trois-Rivières, QC, Canada

**Keywords:** pitchers, strength, skills, training, maturation

## Abstract

Objective assessments of players performance and individual characteristics are increasingly used in baseball. However, evidence linking individual characteristics to players' performance are scarce. The purpose of the study was to identify across ages, in younger males and females, and to compare, in younger males, the anthropometrics, athletic abilities and perceptual-cognitive skills associated with baseball pitcher's ball velocity. A cross-sectional design was used to conduct this study. Male and female athletes completed a sociodemographic questionnaire followed by anthropometric, athletic ability, perceptual-cognitive skill and pitching velocity assessments. Athletes were categorized by their age categories (11U, 13U, 15U, 18U, 21U). To evaluate the athletes' anthropometrics, height and weight, BMI, waist circumference, arms segmental length and girth were measured. Athletic abilities were assessed using athletes' grip strength, upper body power, vertical jump height, sprint, change of direction, and dynamic balance. Perceptual-cognitive skills performance was assessed with the Neurotracker platform. Pitching performance assessment was completed using the athletes' average fastball velocity. Kendall Tau's correlation coefficient was used to assess relationships between variables and pitching velocity in male athletes (*p* < 0.05). A 1-way ANOVA was performed to identify differences between age categories for all variables in male athletes (*p* < 0.05). In male athletes, without age categories discrimination, all anthropometric, athletic ability and perceptual-cognitive skill factors were associated with pitching velocity with associations ranging from τ = 0.185 for perceptual-cognitive skills to τ = 0.653 for left arm grip strength. The results showed that significant differences exist between age categories for anthropometric, athletic ability and perceptual-cognitive skill assessments. The study showed that associations between anthropometrics and pitching velocity, and athletic abilities and pitching velocity vary across age categories. Descriptive data of female athletes results regarding anthropometrics, athletic abilities, perceptual-cognitive skills and pitching velocity are also presented. Gender differences should be investigated in future studies exploring baseball pitching performance.

## Introduction

Baseball is one of the most popular and cherished sports in America. For the 2018–19 academic year, high school amateur baseball players accounted for nearly half a million players when combining male and female players in the United States (NFHS, 2019). Furthermore, there were 36,011 active National Collegiate Athletic Association (NCAA, 2019) college baseball players from division one to division three during the same academic year. These numbers represent only a fraction of college baseball players, as there are also other competitive collegiate baseball associations in the country that would add to this number, such as the National Junior College Athletic Association (NJCAA) and the National Association of Intercollegiate Athletics (NAIA). Over the past two decades, the sport has evolved; one of the major transformations being the widespread use of baseball performance outcome analytics, such as advanced statistics (sabermetrics) and player skill assessment technologies (Healey, 2017; Elitzur, 2020). Numerous assessment technologies are now used alone, or in combination, to assess the biomechanical, physiological, and athletic abilities of baseball players, as well as the baseball flight physics from practice to in-game performance. The use of such technologies is now common in all categories of players, due to increased availability and ease of use. Moreover, statistics and data drawn from biomechanical, physiological, and athletic ability assessment technologies are now used by amateur and professional sports organizations for recruitment (Omoregie, 2016; Parekh and Patel, 2017) to elaborate players' motor skill development strategies while tracking workload (Fadde and Zaichkowsky, 2018; Seshadri et al., 2019) and to prevent injuries (Doeven et al., 2018; Fort-Vanmeerhaeghe et al., 2020). There is no doubt that more information about players' skills and performance can help identify talented players and develop future stars, but there is still one important question remaining; *what are the specific anthropometric, athletic performance and perceptual-cognitive skill factors associated with baseball pitching velocity (PV)*?

Major League Baseball (MLB) pitchers saw their average fastball velocity in miles per hour (MPH) increase from 2008 to 2016 (Castrovince, 2016). The percentage of total pitches registering 95 MPH or more was 4.82% in 2008, compared to 9.14% in 2016 (Castrovince, 2016). There is also evidence of improved in-game performance in MLB pitchers, with a positive and moderate association between average 4-seam fastball velocity and in-game strikeout rate (K%) (r = 0.32), with a minimum of 25 pitching appearances required for qualification (PA) for the 2020 season (MLB, 2021). The identification of clear associations between individual factors such as anthropometrics (Nakata et al., 2013; Papadakis et al., 2021), physical tests (Spaniol, 2009; Lehman et al., 2013; Nakata et al., 2013; Donahue et al., 2018), movement kinetics (Oyama et al., 2013; Reinold et al., 2018) and kinematics (Wang et al., 1995; Werner et al., 2008; Sgroi et al., 2015) and on-field baseball performances remains challenging. In an effort to shed some light on baseball performance characteristics a scoping review synthesizing the individual factors associated with baseball pitching performance was published (Mercier et al., 2020). The review systematically screened publications, extracted data, and assessed risk of bias of all relevant studies investigating the association between individual factors and baseball pitching performance. The review showed that original studies were heterogenous and most often presented moderate to high risks of bias. Results from the review associated with ball velocity showed that kinematics, kinetics, timing outcomes, physical tests, individuals' characteristics, and other individual parameters were found to be potential individual factors associated with pitching performances. Kinematics features such as forward shoulder horizontal adduction and upper torso forward flexion (Escamilla et al., 2002; Stodden et al., 2005; Oyama et al., 2013; Solomito et al., 2018), maximal shoulder external rotation (Escamilla et al., 2002; Chen et al., 2016), upper torso rotation angle and upper torso lateral flexion (Stodden et al., 2001; Oyama et al., 2013; Solomito et al., 2015), lead knee flexion and forward trunk tilt (Matsuo et al., 2001; Stodden et al., 2001, 2005; Escamilla et al., 2002; Dun et al., 2007; Oyama et al., 2013) were found to be significantly associated with increased ball velocity. Kinetics features such as shoulder proximal force and peak elbow proximal force (Stodden et al., 2005; Oyama et al., 2013) were also found to be significantly associated with greater ball velocity. Finally, performances in jumping tests (Lehman et al., 2013; Nakata et al., 2013), medicine ball throw and grip strength (Nakata et al., 2013), players body weight and age (Matsuo et al., 2001; Escamilla et al., 2002; Werner et al., 2008; Lehman et al., 2013; Nakata et al., 2013; Sgroi et al., 2015) were found to be significantly associated with pitching performance and ball velocity. Although several individual factors have been linked to ball velocity, only a few studies investigated such associations in younger athletes. To our knowledge, no study has explored baseball pitching performance predictors in female athletes. Still, evidence about younger athletes seems necessary to optimize athlete development and offer high performance training programs.

To the best of our knowledge, since the scoping review was published in February 2020, five studies related to baseball pitching performance were published. One, a review, reported on workload monitoring of baseball pitchers (Dowling et al., 2020). One study investigated the effect of recovery methods between innings on pitching performance during a simulated game (Wu et al., 2020). Another study focused on the assessment of athletic ability and injury risk related to pitching performance (Mayberry et al., 2020). The fourth study investigated the effect of a hot environment on pitching and hitting in professional baseball players (Huang et al., 2021). Another study investigated upper body contributions to ball velocity in elite high school pitchers using an induced velocity analysis (Alderink et al., 2021). Only the latter study investigated younger baseball pitcher's performance (Alderink et al., 2021).

Given that baseball performance assessment technologies and tools are now being used as recruitment tools by amateur and professional baseball organizations and given the paucity of evidence concerning the association between individual factors and pitching performance, it seems relevant to study individual factors associated with baseball PV in a younger population. The purpose of this study was therefore to identify across ages, in younger male and female athletes, and to compare, in younger male athletes, the anthropometrics, athletic abilities and perceptual-cognitive skills associated with baseball pitchers' ball velocity. We hypothesized that higher scores on athletic performance tests would be associated with higher pitching velocity results for male pitchers. We also hypothesized that anthropometrics and athletic ability tests would be more strongly associated with PV in younger athletes than in older and experienced players.

## Methods

To examine individual factors associated with baseball PV of young male and female athletes, a cross-sectional study design was chosen, and a convenience sampling strategy was used. A quantitative analysis of athletes' baseball PV, anthropometrics, athletic abilities and perceptual-cognitive skills were conducted to investigate the relationship between PV and these individual factors in our population. Baseball PV was assessed using pitcher fastball velocity on a regular mound with distance according to the players' age as per the Baseball Quebec Federation's rulebook (11U = 44 feet, 13U = 48 feet, 15U = 54 feet, 18U and 21U = 60.6 feet) (Baseball Québec, 2021). Anthropometric factors such as height, weight, upper extremity lengths and girth and waist circumference were measured. The assessment of the athletic abilities namely grip strength, upper body power, vertical jump height, sprinting, change of direction, dynamic balance, and perceptual-cognitive skills performance were performed to study their relationship with PV. In this study, these individual factors of baseball pitching performance were used to identify, in male and female baseball pitchers, and compare, in male baseball pitchers the differences across athletes from different age categories.

### Research Sample

Athletes and their parents or tutors were informed of the risks and benefits before providing their written informed consent to participate in this study. The study was approved by the university's local ethics committee (no. CER-20-268-07.25). Male (*n* = 150; 14.28 ± 2.43 years) and female (*n* = 20; 12 ± 1.38 years) baseball players volunteered to participate in this study. Athletes were included if they had at least 1 year of organized baseball experience and were between the age of 10 and 22. Athletes did not need to identify “pitcher” as their primary position, but prior experience on a pitching mound was required to participate in the study. Athletes were also required to be injury free and able to participate in baseball-related activities without any restrictions. Athletes who reported pain while doing the experimentation were excluded from the study. The Baseball Quebec Federation assigns players both by age categories (9U, 11U, 13U, 15U/16UF, 18U/21UF, and 21U) and level of competition (B, A, AA, AAA, Elite) (Baseball Québec, 2021). A sociodemographic questionnaire describing the self-reported player's profile was completed by players and their parents or guardians if they were under 16 years of age, or by the player themselves if they were over 16 years old before testing experimentation. Tables 1, 2 describes the athletes' profiles.

**Table 1:** Descriptions of male and female athletes.

**Categories**	Height (m)^*^	Weight (kg)^*^	BMI (kg/m^2^)^*^	Age^*^	Years of baseball season experience^**^	Years of organized baseball season experience^**^
**Male athletes**						
11U (*n* = 11)	1.45 ± 0.09	41.36 ± 8.59	19.80 ± 3.25	10.55 ± 0.52	5.40 ± 2.01 (*n* = 10)	4.70 ± 1.49 (*n* = 10)
13U (*n* = 54)	1.58 ± 0.08	52.34 ± 12.81	20.7 ± 3.91	12.54 ± 0.50	6.60 ± 2.01 (*n* = 50)	6.48 ± 1.98 (*n* = 50)
15U (*n* = 35)	1.73 ± 0.06	65.98 ± 11.25	22.10 ± 3.14	14.26 ± 0.61	7.69 ± 1.78 (*n* = 29)	7.45 ± 1.68 (*n* = 29)
18U (*n* = 36)	1.77 ± 0.09	73.43 ± 11.33	23.32 ± 2.86	16.42 ± 0.87	10.10 ± 2.41 (*n* = 29)	9.72 ± 2.17 (*n* = 29)
21U (*n* = 14)	1.78 ± 0.05	80.83 ± 9.06	25.58 ± 3.07	18.50 ± 2.68	12.29 ± 2.67 (*n* = 14)	11.86 ± 2.51 (*n* = 14)
Total (*n* = 150)	1.67 ± 0.13	62.45 ± 16.2	22.04 ± 3.07	14.28 ± 2.431	8.12 ± 2.94 (*n* = 132)	7.84 ± 2.80 (*n* = 132)
**Female athletes**						
11U (*n* = 7)	1.46 ± 0.08	41.57 ± 8.54	19.59 ± 4.95	10.57 ± 0.54	5.17 ± 1.72 (*n* = 6)	5.17 ± 1.72 (*n* = 6)
13U (*n* = 11)	1.57 ± 0.07	51.91 ± 5.91	21.00 ± 2.45	12.55 ± 0.93	5.18 ± 2.32 (*n* = 11)	5.18 ± 2.32 (*n* = 11)
15U (*n* = 2)	1.67 ± 0.02	56.00 ± 5.70	20.23 ± 2.56	14.00 ± 0.00	4.00 ± 2.32 (*n* = 2)	4.00 ± 2.32 (*n* = 2)
Total (*n* = 20)	1.54 ± 0.10	48.70 ± 8.57	20.42 ± 3.42	12.00 ± 1.38	5.05 ± 2.01 (*n* = 19)	5.05 ± 2.01 (*n* = 19)

* *Data presented as group mean ± SD*.

** *Data presented as group mean ± SD for available data from subjects who answered the sociodemographic questionnaire*.

**Table 2:** Competitive levels of male and female athletes and school-related sports participation.

Categories^*^	Level of competition %^*^					School's related baseball program participation %^*^		Other sports school's related program participation %^*^	
	**B**	**A**	**AA**	AAA^**^	Elite^***^	**Yes**	**No**	**Yes**	**No**
**Male athletes**									
11U (*n* = 11)	0	0	100	–	–	10 (*n* = 10)	90 (*n* = 10)	10 (*n* = 10)	90 (*n* = 10)
13U (*n* = 50)	24	50	26	–	–	95.74 (*n* = 47)	4.26 (*n* = 47)	2.13 (*n* = 47)	97.87 (*n* = 47)
15U (*n* = 30)	6.7	20	73.3	–	–	86.2 (*n* = 29)	13.8 (*n* = 29)	13.8 (*n* = 29)	86.2 (*n* = 29)
18U (*n* = 32)	0	6.7	26.7	66.6	–	83.3	16.7	3.3	97.7
21U (*n* = 14)	0	0	0	–	100	71.4	28.6	0	100
Total *n* = 137	11	24	39.4	16.1	9.5	82.7 (*n* = 136)	17.3 (*n* = 136)	5.3 (*n* = 136)	94.7 (*n* = 136)
**Female athletes**									
11U (*n* = 7)	86.7	14.3	0	–	–	0	100	14.3	86.7
13U (*n* = 11)	72.7	27.3	0	–	–	27.3	72.7	9.1	89.9
15U (*n* = 2)	50	50	0	–	–	0	100	0	100
Total (*n* = 19)	70	30	–	–	–	15.8	84.2	10.5	89.5

* *Data presented as percentage for available data from subjects who answered the sociodemographic questionnaire*.

** *AAA only exist in the male 18U category*.

*** *Elite only exist in the male 21U category*.

### Procedures

Male and female pitchers were tested once between September 2020 and February 2021. Testing took place during the athlete's off-season in the province of Quebec, Canada at four different facilities depending on the players' baseball program. Before experimentation, the research team ensured that these facilities had the same testing conditions, and that testing protocol could be replicated in each facility. The same evaluators and equipment were used for the entire project. Evaluators were professionally trained kinesiologists with expertise in the strength and conditioning field and athletes graded exercise testing. They were specifically trained for their assigned testing assessment before experimentation. Upon arrival, and prior to the experimentation, athletes followed their usual dynamic body warm-up protocol led by their coach or themselves as they are used to before a regular practice or game. Before the start of the testing protocol, the evaluators asked the athletes if they were ready to perform the protocol at full capacity.

#### Pitching Velocity

PV was used to measure pitching performance. The Rapsodo® Pitching 2.0 system (RP) was used to collect data. This system consists of a monocular camera-based system mounted on the ground 15 feet and 6 inches in front of home plate, in the throwing lane. The RP system provides instantaneous data on pitch velocity, spin rate and axis and ball flight parameters. It has been used in recent studies assessing pitcher performances (Diffendaffer et al., 2019; Lin et al., 2019). Each athlete used a regular 5 oz baseball to assess their PV in this study. Athletes were given 25 pitches to warm up on their own and be ready to throw at full capacity. Then, the velocity of 10 pitches on the pitching mound at full capacity was recorded in MPH. The athletes were asked to throw as accurately as possible, but accuracy was not considered in the results. Athletes were asked to throw either from a stretch or windup position. They were asked to stay consistent for full capacity throws that were recorded for the experimentation. The mean velocity of pitches was used for statistical analysis.

#### Anthropometrics

##### Height, Weight, and Body Mass Index

The height was measured using a portable stadiometer positioned on a level floor against a wall. Athletes were asked to step on the stadiometer barefoot and the measure was taken to the nearest 0.1 cm (Li et al., 2016). Weight was measured with a portable SECA 876 scale that was placed on a non-carpeted hard floor. Athletes were asked to step on the scale barefoot. Weight was taken to the nearest 0.1 kg (Li et al., 2016). Body mass index was calculated with the formula kg/m^2^ (ACSM, 2013).

##### Circumference Measurements and Segmental Length Measurements

The arms (AG) and forearms (FG) girth and waist circumferences (WC) were all measured using a flexible measuring tape to the nearest millimeter. The AG was measured by measuring the largest portion of the arm between the acromion process and the elbow joint. The FG was taken by measuring the widest portion of the forearm between the elbow joint and the radial and ulnar styloid processes (Burkhart et al., 2008). WC was measured superior of both iliac crest in line with the midaxillary line (ACSM, 2013). The lateral arm (LAL) and medial forearm (MFL) length measurements were all taken with a flexible measuring tape to the nearest millimeter. The LAL was taken by measuring the distance between the acromion process and the lateral epicondyle of the elbow joint. The MFL was taken by measuring the distance between the medial aspect of the elbow joint and the distal ulnar styloid process (Burkhart et al., 2008).

#### Athletic Abilities

##### Grip Strength

Grip strength (GP) was measured with a Jamar dynamometer (Mathiowetz, 2002). The device provides a result in kilograms. GP was assessed one arm at a time, in a seated position with both elbows supported on the chair's arms support with both feet on the ground. Athletes were asked to take a deep breath and squeeze the dynamometer handle as hard as possible with their assessed arm and hold it for 3 s. The athletes were given two familiarization trials for each arm. Then, three measures for each arm were taken. Athletes had to alternate between the left and the right arm after each measure for a total of six measures. The mean score of both hands for the three measures was used for statistical analysis.

##### Upper Body Power

The seated medicine ball throw (SMBT) was used to measure upper body power (UBP). The Ballistic Ball (BB) (Assess2Perform, Colorado, USA) was the instrument chosen to measure UBP with the peak velocity value measured in meters per second (m/s). The SMBT protocol was similar to the one described in the Beckham et al. (2019) study, where they investigated test-retest reliability using the Pearson's interclass coefficient (r) and coefficient of variation (CV) of the BB (r = 0.94–0.98; CV % = 4.2–6.8). Athletes performed two familiarization throws with corrections from evaluators if performed incorrectly. Then, three full capacity throws were recorded, and the mean value of the throws was used for statistical analysis.

##### Vertical Jump

The vertical jump (VJ) height test measured the lower body vertical power. The Optojump photoelectric cells (Microgate, Bolzano, Italy) was the instrument used to measure VJ in centimeters. Glatthorn et al. (2011) demonstrated the concurrent validity using the intraclass correlation coefficient (ICC) [ICC = 0.998 (0.995–0.999)] 95% confidence interval (CI) and reliability [ICC = 0.984 (0.960–0.994); CV % = 2.8] of the Optojump instrument compared to a force plate, which is considered the gold standard instrument in terms of measuring VJ. Athletes were instructed to stand between the bars of the Optojump instrument and were told to jump as high as they could, using their arms and legs, and land on both feet in a static and controlled position. The athletes performed two familiarization jumps before three jumps were recorded at full capacity. The mean value of the jumps was used for statistical analysis.

##### 10-m Sprint

Sprint performance was measured with a 10-m sprint using the Brower Timing System (Brower Timing Systems, Utah, USA). This system is an infrared photocell system that uses two electronic sensors positioned on the start and finish line and that expresses the result in seconds. The 10-m sprint protocol was found to be associated with ball velocity in the Nakata et al. (2013) study. Starting in a baserunner position at the starting line, as close as possible from the timing gates, the athletes performed two familiarization sprints, before three sprints at full capacity were recorded. The mean value of the sprints was taken for statistical analysis.

##### Change of Direction

Change of direction was assessed with the pro-agility test (PAT). Stewart et al. (2014) studied this change of direction test and found that it was highly reliable in a pooled sample of male and female physical education students [ICC = 0.88 (0.61–0.88); CV % = 2.4] among other change of direction testing protocols, and that with heterogenous samples, they all measured the same outcome. The athletes had to perform the test as fast as possible using the protocol outlined by Tomchuk (2010). Two familiarization trials, where the athletes did one start from the right side and one start from the left side, were performed before measures were taken. After the familiarization trials, athletes chose their favorite side and made all three of their official trials from the same side. They positioned themselves at the center of the starting line with both feet equally beside the line. The mean value from the three trials was used for statistical analysis.

##### Dynamic Balance

The Y balance test (YBT) was used to assess dynamic balance control. Greenberg et al. (2019) demonstrated an excellent inter-rater reliability between testing days for the YBT composite score for the right limb [Day 1 = ICC = 0.987 (0.972–0.994) 95% CI] and left limb [Day 1 = ICC = 0.982 (0.960–0.992) 95% CI], and a moderate to excellent test-retest reliability for both right [ICC = 0.810 (0.672, 0.893) 95% CI] and left YBT composite score [ICC = 0.793 (0.639, 0.885) 95% CI]. YBT testing protocol was the same used in Greenberg et al. (2019) study, but with the use of the Movement Assessment Technology (MAT) as the instrument tool. Dynamic balance was assessed barefoot by three directional reaches per foot (anterior, right posteromedial, left posteromedial), while maintaining a single-leg stance. The composite score, the sum of the three directional reaches, was used for statistical analysis.

#### Perceptual-Cognitive Skills

The perceptual-cognitive skills of athletes were assessed using the Neurotracker platform (Cognisens, Canada), which consists of a three-dimensional multiple object tracking (3D-MOT) task displayed on a large visual field (Faubert and Sidebottom, 2012). This tool has been shown to correlate with perceptual skills in various populations (Mangine et al., 2014; Harenberg et al., 2016; Lysenko-Martin et al., 2020), including athletes (Mangine et al., 2014). In this study, the athletes sat in a dark room in front of a screen projected by a 3D projector while wearing active 3D glasses. Using the *CORE* mode on the Neurotracker platform, the athletes were asked to visually follow 4 specific spheres while they moved for 8 s among distractors in the 3D virtual space displayed by the projector. A total of 20 trials (8 s per trial) were performed during which a staircase calculation ran by the program adjusted maximal speed at which participants were able to adequately follow the 4 spheres (i.e., speed was increased after a successful trial, or decreased after an unsuccessful trial). The athletes manually entered their answers after each individual trial on a wireless keyboard. The performance achieved over the 20 trials represents the speed (meters per second) at which an individual can adequately perform the task and is referred to as the *Speed Threshold*. Before undergoing testing, athletes were provided a 5-trial practice. The speed threshold score was used for statistical analysis.

##### Statistical Analysis

Descriptive data including mean ± SD and percentages of individual characteristics were calculated. Normal distribution was assessed using the Shapiro-Wilks test and visual inspection. A 1-way analysis of variance (ANOVA) was performed to identify differences between age categories for all studied variables of male athletes. Tukey's *post-hoc* analyses were then conducted to identify specific group differences among age categories. Because the primary variable PV was not normally distributed, non-parametric statistical procedures were used for correlations. The Kendall-tau (τ) correlation was used to compute and assess correlation between each individual factor and PV for the total sample (male) and by age categories (male). Because of the small number of female athletes recruited, only descriptive data are presented. The significance levels for all analyses were set to *p* < 0.05. Statistical computations were performed using IBM SPSS Statistics version 27.0.1.

## Results

**Figure 1:**
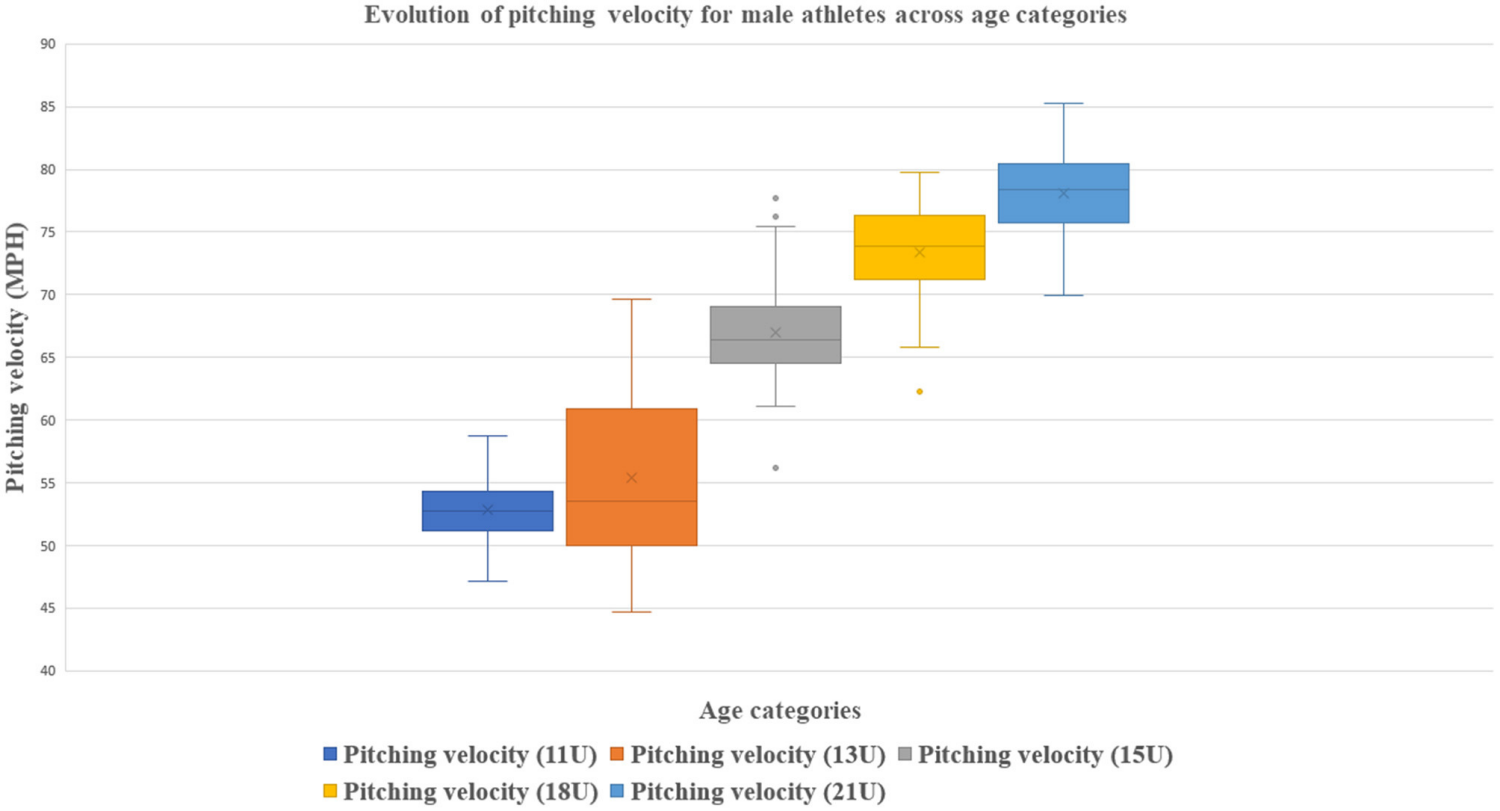
Evolution of pitching velocity across age categories for male athletes.

**Figure 2:**
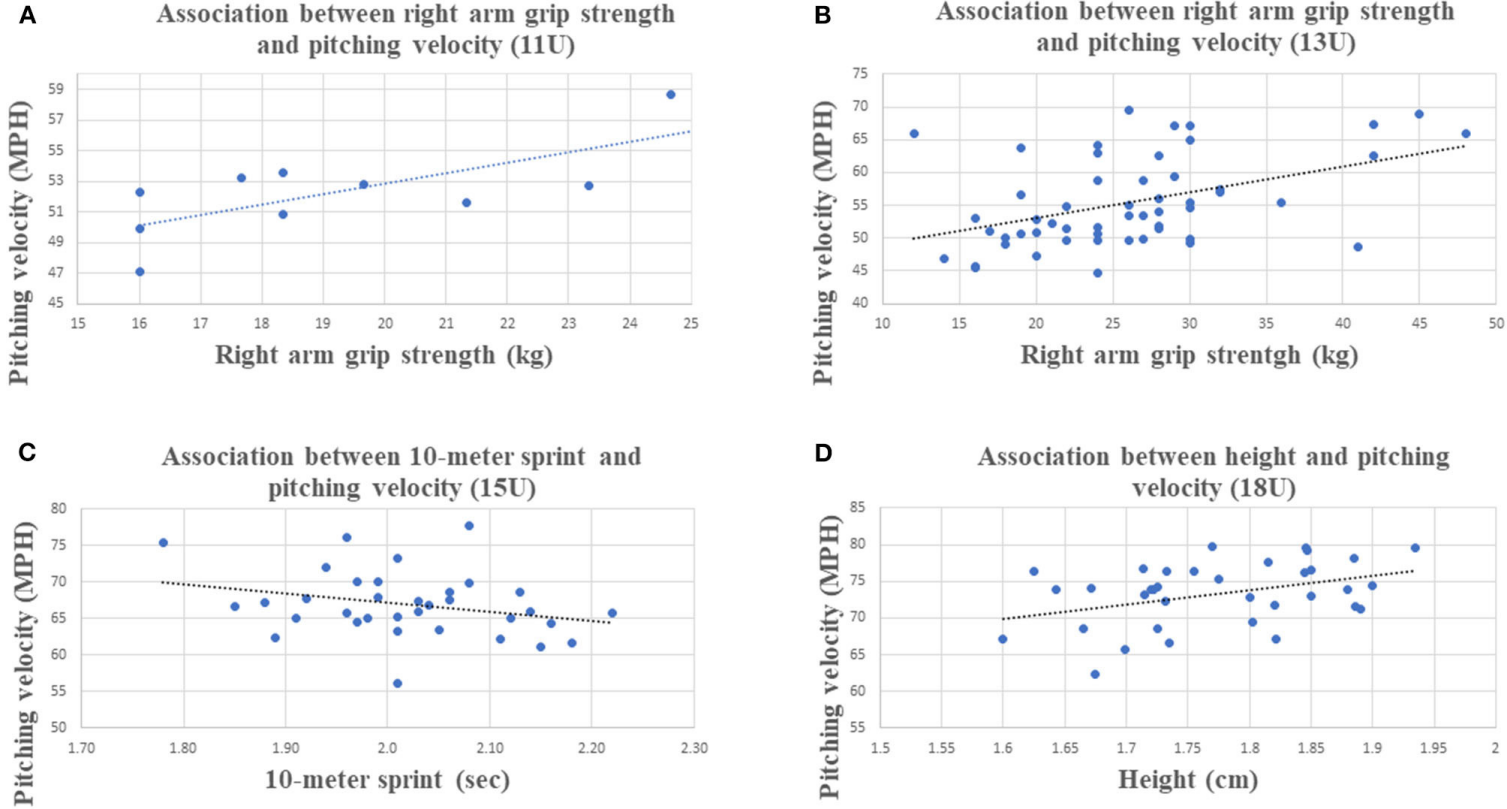
Strongest association with PV by age categories. **(A)** Association between right arm grip strength and pitching velocity (11U). **(B)** Association between right arm grip strength and pitching velocity (13U). **(C)** Association between 10-m sprint and pitching velocity (15U). **(D)** Association between height and pitching velocity (18U).

The 1-way ANOVA for male athletes showed significant differences across age categories (*p* < 0.05) for all studied variables. Table 3 shows descriptive means and standard deviations of anthropometrics, athletic abilities and perceptual cognitive skills for male athletes across age categories and total sample. Table 4 shows descriptive means and standard deviations of anthropometrics, athletic abilities and perceptual cognitive skills for female athletes across age categories and total sample. Table 5 shows the 1-way ANOVA with CI. Figure 1 shows pitching velocity evolution across age categories for male athletes whereas Figure 2 shows strongest association with PV by age categories.

**Table 3:** Results of anthropometric, athletic ability, and perceptual-cognitive skill assessments of male athletes.

	**Group**	***N***	**Mean**	**±SD**
**Anthropometrics**				
Height (m)	11U	11	1.45	0.09
	13U	54	1.58	0.08
	15U	35	1.73	0.06
	18U	36	1.77	0.09
	21U	14	1.78	0.05
	Total	150	1.67	0.12
Weight (kg)	11U	11	41.64	8.59
	13U	54	52.32	12.82
	15U	35	65.98	11.26
	18U	36	73.43	11.33
	21U	14	80.83	9.06
	Total	150	62.45	16.20
BMI (kg/m^2)^	11U	11	19.80	3.25
	13U	54	20.70	3.91
	15U	35	22.08	3.14
	18U	36	23.32	2.86
	21U	14	25.58	3.06
	Total	150	22.04	3.70
Waist circumference (cm)	11U	11	70.05	6.12
	13U	54	73.37	9.28
	15U	35	77.29	8.27
	18U	36	80.21	7.24
	21U	14	85.29	9.25
	Total	150	76.80	9.26
Right arm girth (cm)	11U	11	22.59	3.43
	13U	53	25.77	3.53
	15U	35	28.25	3.19
	18U	36	30.19	2.86
	21U	14	33.18	2.41
	Total	149	27.88	4.20
Left arm girth (cm)	11U	11	22.68	3.23
	13U	53	25.59	3.63
	15U	35	27.67	2.59
	18U	36	29.82	2.73
	21U	14	32.68	2.20
	Total	149	27.56	3.99
Right forearm girth (cm)	11U	11	21.38	2.50
	13U	53	24.45	2.93
	15U	35	25.87	1.67
	18U	36	27.08	2.13
	21U	14	28.39	1.16
	Total	149	25.56	2.89
Left forearm girth (cm)	11U	11	21.23	2.13
	13U	53	24.28	2.84
	15U	35	25.67	1.71
	U18	36	26.75	2.02
	21U	14	27.86	0.95
	Total	149	25.31	2.76
Right lateral arm length (cm)	11U	11	26.00	1.77
	13U	53	31.96	3.55
	15U	35	33.83	2.58
	18U	36	35.33	3.74
	21U	14	37.50	3.44
	Total	149	33.30	4.23
Left lateral arm length (cm)	11U	11	26.50	1.52
	13U	53	31.73	3.57
	15U	35	33.89	2.77
	18U	36	35.61	3.71
	21U	14	37.64	3.66
	Total	149	33.34	4.28
Right medial forearm length (cm)	11U	11	20.68	1.57
	13U	53	24.88	2.43
	15U	35	26.14	1.66
	18U	36	27.19	1.78
	21U	14	27.57	1.64
	Total	149	25.68	2.63
Left medial forearm length (cm)	11U	11	20.82	1.74
	13U	53	24.76	2.59
	15U	35	26.30	1.83
	18U	36	27.11	1.86
	21U	14	27.68	1.50
	Total	149	25.67	2.72
**Athletic abilities**				
Right arm grip strength (kg)	11U	11	19.70	3.48
	13U	54	25.97	7.57
	15U	35	35.91	6.31
	18U	36	44.92	9.25
	21U	14	50.50	9.67
	Total	150	34.66	12.32
Left arm grip strength (kg)	11U	11	19.24	4.22
	13U	54	24.69	7.08
	15U	35	36.19	6.40
	18U	36	42.99	8.39
	21U	14	47.74	6.68
	Total	150	33.52	11.60
Vertical jump height (cm)	11U	11	28.52	2.99
	13U	54	35.49	6.21
	15U	35	42.54	7.58
	18U	36	46.87	7.34
	21U	14	48.54	8.81
	Total	150	40.57	9.13
10-m sprint (cm)	11U	11	2.31	0.11
	13U	54	2.19	0.14
	15U	35	2.02	0.10
	18U	36	1.96	0.09
	21U	14	1.91	0.11
	Total	150	2.08	0.17
Pro-agility test (cm)	11U	11	5.79	0.40
	13U	54	5.57	0.40
	15U	35	5.15	0.26
	18U	36	5.14	0.29
	21U	14	4.98	0.25
	Total	150	5.33	0.41
Upper body power (m/s)	11U	10	2.51	0.29
	13U	53	2.94	0.49
	15U	35	3.78	0.44
	18U	36	4.55	0.74
	21U	14	5.54	0.72
	Total	148	3.75	1.05
YBT composite score right leg (cm)	11U	11	170.79	18.58
	13U	54	214.28	20.32
	15U	35	231.75	17.52
	18U	36	245.42	16.04
	21U	14	245.10	21.30
	Total	150	225.52	27.35
YBT composite score left leg (cm)	11U	11	172.79	15.37
	13U	54	218.94	19.90
	15U	35	233.33	16.30
	18U	36	248.80	16.47
	21U	14	246.17	18.97
	Total	150	228.62	26.63
**Perceptual-cognitive skills**				
Neurotracker (m/s)	11U	11	0.88	0.33
	13U	25	1.09	0.52
	15U	19	0.98	0.32
	18U	13	1.41	0.55
	21U	3	0.94	0.24
	Total	71	1.08	0.47
**Pitching velocity**				
Pitching velocity (MPH)	11U	11	52.60	3.15
	13U	53	55.40	6.79
	15U	34	66.96	4.45
	18U	35	73.36	4.31
	21U	14	78.10	3.69
	Total	147	64.30	10.28

**Table 4:** Results of anthropometric, athletic ability, and perceptual-cognitive skill assessments of female athletes.

	**Group**	***N***	**Mean**	**±SD**
**Anthropometrics**				
Height (m)	11U	7	1.46	0.08
	13U	11	1.57	0.07
	15U	2	1.67	0.02
	Total	20	1.54	0.10
Weight (kg)	11U	7	41.57	8.54
	13U	11	51.91	5.91
	15U	2	56.00	5.66
	Total	20	48.70	8.56
BMI (kg/m^2)^	11U	7	19.59	4.95
	13U	11	21.00	2.45
	15U	2	20.23	2.56
	Total	20	20.43	3.42
Waist circumference (cm)	11U	7	65.09	9.53
	13U	11	68.10	6.26
	15U	2	69.00	5.66
	Total	20	67.13	7.31
Right arm girth (cm)	11U	7	22.64	3.45
	13U	11	24.27	2.17
	15U	2	24.75	1.77
	Total	20	23.75	2.66
Left arm girth (cm)	11U	7	22.57	3.35
	13U	11	24.10	1.87
	15U	2	24.25	2.47
	Total	20	23.58	2.50
Right forearm girth (cm)	11U	7	21.86	2.28
	13U	11	22.50	1.38
	15U	2	23.25	0.35
	Total	20	23.35	1.69
Left forearm girth (cm)	11U	7	21.64	2.14
	13U	11	22.41	1.18
	15U	2	23.00	0.71
	Total	20	22.20	1.55
Right lateral arm length (cm)	11U	7	26.58	1.51
	13U	11	29.64	1.69
	15U	2	20.25	1.06
	Total	20	29.08	1.95
Left lateral arm length (cm)	11U	7	26.21	1.15
	13U	11	28.60	1.22
	15U	2	30.50	1.41
	Total	20	27.95	1.82
Right medial forearm length (cm)	11U	7	21.64	1.14
	13U	11	23.31	1.37
	15U	2	25.50	2.12
	Total	20	22.95	1.74
Left medial forearm length (cm)	11U	7	21.79	1.07
	13U	11	23.18	1.40
	15U	2	25.25	1.77
	Total	20	22.90	1.62
**Athletic abilities**				
Right arm grip strength (kg)	11U	7	18.05	3.31
	13U	11	21.21	4.27
	15U	2	29.67	4.24
	Total	20	20.85	4.84
Left arm grip strength (kg)	11U	7	16.81	3.06
	13U	11	20.58	3.53
	15U	2	29.33	5.66
	Total	20	20.13	4.93
Vertical jump height (cm)	11U	7	29.55	4.86
	13U	10	29.76	4.54
	15U	2	31.52	11.01
	Total	19	30.29	5.37
10-m sprint (cm)	11U	7	2.32	0.16
	13U	10	2.24	0.12
	15U	2	2.10	0.15
	Total	19	2.25	0.15
Pro-agility test (cm)	11U	7	6.10	0.47
	13U	10	5.80	0.22
	15U	2	5.80	0.23
	Total	19	5.88	0.43
Upper body power (m/s)	11U	6	2.26	0.17
	13U	10	2.58	0.32
	15U	2	2.89	0.10
	Total	18	2.51	0.32
YBT composite score right leg (cm)	11U	7	201.71	16.61
	13U	11	203.88	69.10
	15U	2	241.00	24.98
	Total	20	206.83	52.63
YBT composite score left leg (cm)	11U	7	205.48	18.93
	13U	11	207.21	70.05
	15U	2	231.00	28.28
	Total	20	208.98	52.87
**Perceptual-cognitive skills**				
Neurotracker (m/s)	11U	7	0.78	0.33
	13U	11	0.79	0.27
	15U	2	0.76	0.38
	Total	20	0.78	0.28
**Pitching velocity**				
Pitching velocity (MPH)	11U	7	39.07	3.72
	13U	10	46.95	5.40
	15U	2	53.15	7.57
	Total	19	44.70	6.74

**Table 5:** 1-way ANOVA for male athletes.

**1-way ANOVA male athletes**		**Sum of squares**	DF^*^	**Mean square**	***F*-value**	***P*-value**	**95% confidence interval**	
							**Lower**	**Upper**
Height (m)	Between groups	1.600	4	0.400	70.845	<0.001	0.565	0.717
	Within groups	0.819	145	0.006				
	Total	2.418	149					
Weight (kg)	Between groups	19808.395	4	4952.099	37.182	<0.001	0.382	0.583
	Within groups	19312.099	145	133.187				
	Total	39120.493	149					
BMI (kg/m^2^)	Between groups	387.087	4	96.772	8.464	<0.001	0.071	0.281
	Within groups	1657.776	145	11.433				
	Total	2044.863	149					
Waist circumference (cm)	Between groups	2573.637	4	643.409	9.139	<0.001	0.081	0.294
	Within groups	10208.850	145	70.406				
	Total	12782.487	149					
Right arm girth (cm)	Between groups	1133.447	4	283.362	27.653	<0.001	0.302	0.519
	Within groups	1475.601	144	10.247				
	Total	2609.048	148					
Left arm girth (cm)	Between groups	1017.554	4	254.389	27.306	<0.001	0.299	0.516
	Within groups	1341.516	144	9.316				
	Total	2359.070	148					
Right forearm girth (cm)	Between groups	455.370	4	113.843	21.055	<0.001	0.234	0.459
	Within groups	778.592	144	5.407				
	Total	1233.962	148					
Left forearm girth (cm)	Between groups	409.161	4	102.290	20.487	<0.001	0.227	0.453
	Within groups	718.980	144	4.993				
	Total	1128.140	148					
Right arm lateral length (cm)	Between groups	1086.611	4	271.653	25.134	<0.001	0.277	0.498
	Within groups	1556.396	144	10.808				
	Total	2643.007	148					
Left arm lateral length (cm)	Between groups	1107.948	4	276.987	24.912	<0.001	0.275	0.496
	Within groups	1601.096	144	11.119				
	Total	2709.044	148					
Right medial forearm length (cm)	Between groups	448.935	4	112.234	28.256	<0.001	0.308	0.524
	Within groups	571.977	144	3.972				
	Total	1020.911	148					
Left medial forearm length (cm)	Between groups	448.479	4	112.120	25.074	<0.001	0.277	0.497
	Within groups	643.907	144	4.472				
	Total	1092.386	148					
Right arm grip strength (kg)	Between groups	13895.676	4	3473.919	57.772	<0.001	0.508	0.677
	Within groups	8719.013	145	60.131				
	Total	22614.689	149					
Left arm grip strength (kg)	Between groups	12766.029	4	3191.507	63.628	<0.001	0.535	0.696
	Within groups	7272.991	145	50.159				
	Total	20039.019	149					
Vertical jump height (cm)	Between groups	5444.764	4	1361.191	28.265	<0.001	0.307	0.522
	Within groups	6982.872	145	48.158				
	Total	12427.636	149					
10-m sprint (s)	Between groups	2.210	4	0.553	40.440	<0.001	0.406	0.601
	Within groups	1.981	145	0.014				
	Total	4.191	149					
Pro-agility test (s)	Between groups	9.473	4	2.368	21.531	<0.001	0.238	0.462
	Within groups	15.948	145	0.110				
	Total	25.421	149					
Upper body power (m/s)	Between groups	117.567	4	29.392	92.094	<0.001	0.637	0.767
	Within groups	45.638	143	0.319				
	Total	163.205	147					
YBT composite score right leg (cm)	Between groups	60744.824	4	15186.206	43.456	<0.001	0.426	0.617
	Within groups	50671.517	145	349.459				
	Total	111416.341	149					
YBT composite score left leg (cm)	Between groups	59094.146	4	14773.536	46.011	<0.001	0.443	0.629
	Within groups	46557.417	145	321.086				
	Total	105651.562	149					
Neurotracker (m/s)	Between groups	2.140	4	0.535	2.663	0.040	0.000	0.254
	Within groups	13.254	66	0.201				
	Total	15.394	70					
Pitching velocity (MPH)	Between groups	11478.350	4	2869.588	102.958	<0.001	0.666	0.787
	Within groups	3957.732	142	27.871				
	Total	15436.082	146					

* *DF, Degree of freedom*.

### Correlations Between PV and Anthropometrcics Variables, PV and Athletic Abilities, and PV and Perceptual Cognitive Skills for Male Athletes

#### Total Sample

For the male athletes' total sample, correlations between PV and anthropometric variables were all found to be significant (*p* < 0.05) ranging from τ = 0.332 for BMI to τ = 0.617 for height. PV and athletic ability variables were all found to be significantly correlated (*p* < 0.05) ranging from τ = 0.403 for left leg YBT composite score to τ = 0.653 for left arm GP. The correlation between PV and the assessment of perceptual-cognitive skills was τ = 0.185.

#### 11U

In the 11U age category, there was no significant correlation for PV and anthropometric variables. For PV and athletic abilities, right arm GP was the only significant correlation found with a τ = 0.506 (*p* = 0.046) 95% CI [0.028, 0.976]. No significant correlation was found for PV and perceptual-cognitive skills assessment.

#### 13U

In the 13U age category, for PV and anthropometric variables, height τ = 0.473 (*p* < 0.001) 95% CI [0.294, 0.587], weight τ = 0.363 (*p* < 0.001) 95% CI [0.193, 0.512] and WC τ = 0.194 (*p* = 0.043) 95% CI [0.011, 0.364] were found to be significant correlations. For PV and athletic abilities, right arm GP τ = 0.527 (*p* < 0.001) 95% CI [0.381, 0.647], left arm GP τ = 0.500 (*p* < 0.001) 95% CI [0.349, 626], VJ τ = 0.233 (*p* = 0.014) 95% CI [0.052, 0.399], 10-m sprint τ = −0.310 (*p* = 0.001) 95% CI [−00.466, −00.134], PAT τ = −0.201 (*p* = 0.034) 95% CI [−00.371, −00.019] and UBP τ = 0.483 (*p* < 0.001) 95% CI [0.184, 0.508] were found to be significantly correlated. No significant correlation was found for PV and perceptual-cognitive skills assessment.

#### 15U

In the 15U category, for PV and anthropometric variables, there was no significant correlation. For PV and athletic abilities, the 10-m sprint was the only significant correlation found, with a τ = −0.375 (*p* = 0.002) 95% CI [−0.558, −0.156]. No significant correlation was found for PV and perceptual-cognitive skills assessment.

#### 18U

In the 18U category, for PV and anthropometric variables, height τ = 0.352 (*p* = 0.003) 95% CI [0.135, 5.38] and right MFL τ = 0.292 (*p* = 0.018) 95% CI [0.068, 0.488] were found to be significantly correlated. For PV and athletic abilities, left arm GP τ = 0.294 (*p* = 0.014) 95% CI [0.070, 0.489], UBP τ = 0.296 (*p* = 0.013) 95% CI [−0.049, 0.394] and right leg composite YBT τ = 0.260 (*p* = 0.029) 95% CI [0.034, 0.462] were found to be significantly correlated. No significant correlation was found for PV and perceptual-cognitive skills assessment.

#### 21U

In the 21U age category, there was no significant correlation for PV and anthropometric variables, PV and athletic ability variables and PV and perceptual-cognitive skills assessment.

## Discussion

The purpose of this study was to identify across ages, in younger male and female athletes, and to compare, in younger male athletes, the anthropometrics, athletic abilities and perceptual-cognitive skills associated with baseball pitcher's ball velocity. The results showed that significant differences exist in male athletes across age categories (11U, 13U, 15U, 18U, and 21U) for anthropometric, athletic ability and perceptual-cognitive skill assessments. Moreover, when studying associations between anthropometrics, athletic abilities and perceptual-cognitive skills with PV in a sample of male athletes aged 10–22 years (*n* = 150), all variables were found to be associated with PV. Nakata et al. (2013) reported similar results showing that their anthropometrics and athletic abilities were all found to be associated with kinetic energy of pitched ball. They found strong correlation for right (*r* = 0.906) and left (*r* = 0.894) GP, age (*r* = 0.880), height (*r* = 0.870), back strength (*r* = 0.866), and standing long jump (*r* = 0.850), all significant *p* < 0.01. In addition, in this study, multiple regression analyses showed that age, BMI, 10-m sprints, GP and standing long jump were predictors of pitching performance in a sample of young male athletes age 6.4–15.6 years. One must note that although similar results were found, the present study investigated PV with athletes throwing from a pitching mound in a pitcher's specific stretch or windup delivery compared to only throwing the baseball into a net from a standardized distance. Furthermore, the present study investigated older players, using a standardized 5 oz baseball for every subject, with only the distance of the pitching mound and home plate being adjusted for the players' age categories. Considering perceptual-cognitive skills assessment, although a significant association between perceptual-cognitive skills and PV was found, the relationship between this factor and ball velocity was the weakest among all investigated variables (τ = 0.185) in our male sample aged 10–22 years old. Nowadays, multiple commercial perceptual-cognitive skills technologies can be used by sports organizations to track athletes' development (Fadde and Zaichkowsky, 2018). To the authors knowledge, no other studies investigated the relationship between PV and perceptual-cognitive skills in baseball pitchers. One study investigated sensorimotor abilities in professional pitchers to predict their on-field performance using the Fielding-Independent Pitching (FIP) sabermetric (Burris et al., 2018). They found that sensorimotor abilities did not predict the FIP sabermetric and that sensorimotor abilities must be unrelated to pitchers' abilities to perform in game situation. It must be pointed out that PV and on-field performances can only partially explain baseball pitching performance as ball accuracy factors and ball flight parameters should also be considered important factors of pitching performance. Future studies should investigate the relationship between ball accuracy, ball flight parameters and the perceptual-cognitive skills assessment.

Investigating specific associations within each age category showed that different anthropometrics and athletic abilities were found to be associated with PV at different ages. The perceptual-cognitive skills assessment was not associated with PV within any age category. The 13U and 18U categories for male athletes were the categories with the most associations with PV. These different results between associations in our total sample compared to associations within age category between anthropometrics and PV, and athletic abilities and PV could be explained by the dynamic process that is athlete development and the interindividual variability in the maturation process (Malina et al., 2015). Our results showed that the 13U was the age category for which the most associations between anthropometrics and PV, and athletic abilities and PV were found. The highest standard deviation in 12 studied variables (weight, BMI, WC, right and left arm girth, right and left forearm girth, right and left medial forearm length, 10-m sprint, PAT, and PV) were also found in this age category compared to the other age categories investigated in this study. This age category also had the biggest sample size among our age categories (*n* =54), suggesting important differences in physical maturation of the participants. Other studies from different sports also showed that, for 11–13 years old athletes, variability in physical maturation across athletes of this age exists, and that more mature and bigger athletes had higher scores for anthropometric and functional capacity results related to their physical maturation (Malina et al., 2004; Silva et al., 2010).

In other age groups, Lehman et al. (2013) studied correlations between throwing velocity and lower-body field tests in male college baseball players. They found that, for pitchers in a stretch position, lateral to medial jump and body weight explained ~32.2% of the variance in right-handed throwing velocity, and that ~68.8% of the variance in left-handed throwing velocity was explained by the lateral to medial jump. Their study sample (*n* = 42; 19.8 ± 1.2 years) had a similar age range to our 21U group (*n* = 14; 18.50 ± 2.68 years). However, our study did not find associations between the studied variables and PV for this age category.

### Limitations

This study is not without limitations. Competition levels in each category were not accounted for in this study, as athletes were only categorized by their age and not their competition level. Also, the fact that athletes were categorized by their aged instead of their maturation stage potentially influenced our results. Furthermore, the pitching protocol required athletes to perform pitching on the pitching mound for protocol standardization even though participants did not need to identify “pitcher” as their primary position, as mentioned earlier. However, Nissen et al. (2013) found significant differences in kinematics and kinetics on a flat ground vs. a mound in adolescent baseball pitchers between these two pitching position (Nissen et al., 2013; Bullock et al., 2021) found that despite higher velocity for all pitches type, players identified as pitchers had overall similar kinematics and kinetics to players identified as non-pitchers Due to low number of available female athletes, only descriptive analyses were conducted for the different variables.

## Conclusion

The study showed that associations between anthropometrics and PV, and athletic abilities and PV vary across age categories and that in a sample of male athletes, aged 10–22 years old, anthropometric, athletic ability and perceptual-cognitive skill factors were all associated with PV. Gender differences should be investigated, and future studies should therefore explore associations between individual characteristics and performance to establish sex-specific differences.

## Data Availability Statement

The raw data supporting the conclusions of this article will be made available by the authors, without undue reservation.

## Ethics Statement

The studies involving human participants were reviewed and approved by the Comité d'éthique de la recherche avec des êtres humains, Université du Québec à Trois-Rivières (no. CER-20-268-07.25). Written informed consent to participate in this study was provided by the participants' legal guardian/next of kin.

## Author Contributions

MT, CT, and MD participated in data collection. MT, CT, L-AC-B, and MD wrote the manuscript. All authors contributed to the final manuscript revision, read, and approved the submitted version.

## Funding

This study was supported by MITACS (Mitacs accélération, ref.IT19020) and Baseball 360 Trois-Rivières.

## Conflict of Interest

The authors declare that the research was conducted in the absence of any commercial or financial relationships that could be construed as a potential conflict of interest.

## Publisher's Note

All claims expressed in this article are solely those of the authors and do not necessarily represent those of their affiliated organizations, or those of the publisher, the editors and the reviewers. Any product that may be evaluated in this article, or claim that may be made by its manufacturer, is not guaranteed or endorsed by the publisher.
